# The Impact of Preoperative Hepatic Impairment on the Development of Postoperative Delirium: A Systematic Review and Meta-Analysis

**DOI:** 10.7759/cureus.94298

**Published:** 2025-10-10

**Authors:** Will S Roberts, Vaibhav Mittal, Michael N Ryan, Alexander D Knight, Sarah Schaffer, Andrew Clifford, Jean-Pierre P Ouanes

**Affiliations:** 1 Osteopathic Medicine, Nova Southeastern University Dr. Kiran C. Patel College of Osteopathic Medicine, Clearwater, USA; 2 Anesthesiology, Weill Cornell Medicine, New York, USA; 3 Anesthesiology, Hospital for Special Surgery, West Palm Beach, USA

**Keywords:** alcohol consumption, he: hepatic encephalopathy, hepatic impairment, postoperative delirium, surgical outcome

## Abstract

Postoperative delirium (POD) is one of the most common psychiatric and neurologic complications experienced by patients undergoing general anesthesia. While it is widely hypothesized that hepatic impairment is associated with POD, the increased risk that various forms of preoperative hepatic impairment contribute to the development of POD is not well defined in the literature. A database search was performed on Embase, Web of Science, and MEDLINE-PubMed searching for articles containing keywords regarding the association between hepatic impairment and POD, ultimately yielding 34 studies for inclusion. Adjusted odds ratios (ORs) were extracted, with two-sided p-values <0.05 deemed significant. A total of 12,089 patients were included in this review, of which 2,663 developed POD (overall incidence of 22.03%). Inverse variance random-effects meta-analysis reveals that history of alcohol consumption (OR: 2.59, 1.85 - 3.64, p<0.0001), history of hepatic encephalopathy (OR: 3.42, 2.25 - 5.19, p<0.0001), and a MELD (Model for End-Stage Liver Disease) score ≥15 (OR 3.73, 1.93 - 7.20, p<0.0001) are independent risk factors for POD. Elevated serum IL-6 and total bilirubin did not reach significance as risk factors. Based on our findings, a medical history of known hepatic pathology potentially increases a patient’s risk for POD. However, our study did not implicate any specific biomarkers as predisposing risk factors for POD but rather their indicated summative hepatic influence in the form of a MELD score.

## Introduction and background

The global incidence of hepatic-related deaths has been continually on the rise: from 110.6 per 100,000 in 1990 to 132.5 per 100,000 in 2017 [1]. There is a growing population of patients living with some form of hepatic disease and awaiting a liver transplant. In 2021, there was a 6.5% increase compared to the previous year in the number of liver transplants performed globally [1]. There are several indications for patients to receive a liver transplant, including viral hepatitis, non-alcoholic fatty liver disease, hepatocellular carcinoma, and alcoholic hepatitis; with many of these disease processes increasing in incidence [2,3]. Furthermore, patients with any form of liver disease often have comorbidities or complications that may require surgical intervention in addition to requiring a liver transplant. With the increased need for surgery among hepatically impaired patients, postoperative delirium (POD) is a common complication to be aware of.

The risk for POD is further heightened in patients with pre-existing health conditions compared to a healthier population. Diabetes and cardiovascular conditions have been well-established risk factors for POD [4]. The increased surgical risk and care complexities contribute to higher healthcare costs, as well as increase morbidity and negative outcomes among liver disease patients. Despite the growing prevalence of liver disease patients, there is limited research on methods to screen for the risk of POD. Alcohol abuse is a well-documented risk factor for POD [4]. However, there are limited studies on hepatic pathologies as a risk factor. There are multiple biomarkers used to indicate the presence and potential severity of hepatic impairment. In addition to biomarkers, Child-Pugh scores and MELD (Model for End-Stage Liver Disease) are widely used metrics, which combine a variety of factors, such as total bilirubin, serum albumin, international normalized ratio, and serum creatinine, among others, to evaluate patients with cirrhosis [2].

A significant history of alcohol consumption has been previously implicated in the development of POD, among other surgical complications [5]. It is widely known to stem from a multifactorial etiology, and that alcohol can affect several organs, not just the liver. It is hypothesized that the association between alcohol consumption and POD stems from alcohol's disruption of acetylcholine and dopamine levels [6,7]. Alcohol has been linked to an elevation of dopamine levels, which has subsequently been linked to POD [8]. Also, alcohol has been implicated in increased acetylcholine levels [9]. Additionally, several pharmacological agents utilized during general anesthesia undergo major hepatic elimination pathways. Inhibition of these pathways could theoretically lead to overaccumulation of metabolites, especially opioid metabolites that can cross the blood-brain barrier [10]. Alcohol consumption in severe cases can also result in delirium as a function of thiamine depletion and Wernicke encephalopathy [11]. Lastly, a withdrawal from alcohol can precipitate delirium and delirium tremens, as patients are likely to deviate from their normal alcohol consumption leading up to surgery [12].

The objective of this systematic review and meta-analysis was to identify the role that specific indicators of preoperative hepatic impairment contribute to increased risk for POD. We aimed to identify the incidence rate of POD in different surgical populations. To the knowledge of the authors of this review, there are no similar reviews in the literature that address this research topic.

## Review

Materials and methods

Review Registration

The review was registered in PROSPERO under the identification number CRD42024627855..

Database Search and Screening

**Figure 1:**
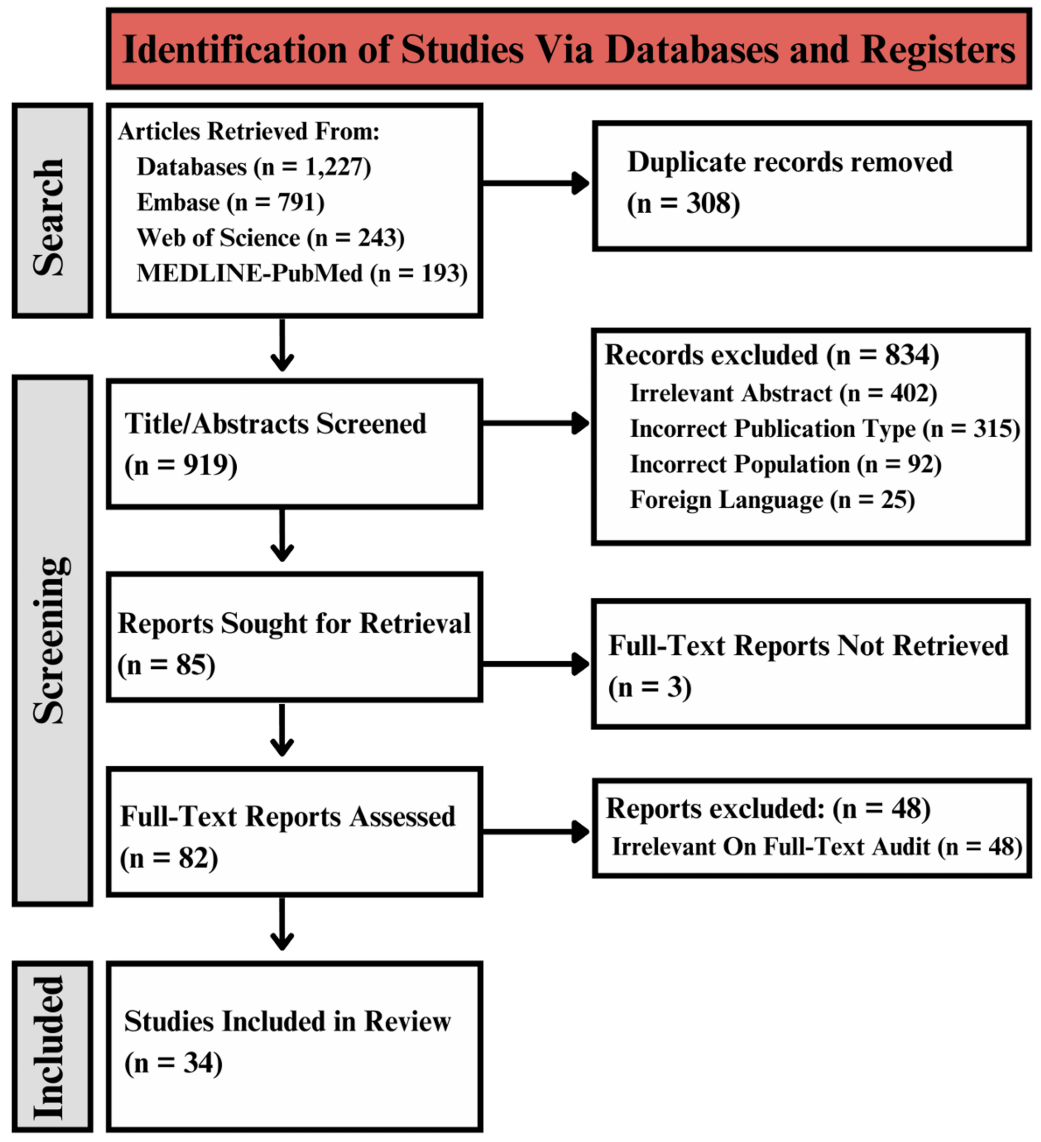
The PRISMA diagram depicting the selection of studies PRISMA: The Preferred Reporting Items for Systematic Reviews and Meta-Analyses

Embase, Web of Science, and OVID-MEDLINE databases were searched for relevant keywords including: aspartate aminotransferase (AST), alanine aminotransferase (ALT), alkaline phosphatase (ALP), gamma-glutamyl transferase (GGT), alpha-fetoprotein (AFP), ceruloplasmin, nucleotidase (NTP), bilirubin, cirrhosis, alcohol, alcoholic, hepatitis, hepatocellular carcinoma (HCC), hemochromatosis and the resulting incidence of POD. Studies isolated from the databases were subjected to a standardized screening process in Rayyan that required the fulfillment of the following metrics: 1. English language, 2. randomized controlled trial, retrospective observational, prospective observational, or case-control study designs, 3. adult, human population, 4. full-text availability, and 5. reporting of a multivariate odds ratio (OR) for the association of aforementioned metrics of hepatic impairment and the incidence of POD (Figure 1) [13]. This search was carried out entirely on December 15th, 2024.

Quality Assessment of Included Studies

All studies included in this review underwent a standardized quality assessment consisting of five independent metrics: the tool used to screen for delirium, at least 100 patient population size, OR standard error less than or equal to 0.5, presence of indirectness, and any evidence of other limitations (Table 1). If a study met all of these criteria, then it was considered of “high” quality for this review. If a study only met four or three of these criteria, then it was considered of “moderate” quality for this review. Lastly, if a study met less than three of these criteria, it was considered of “low” quality for this review.

**Table 1:** Quality assessment of included studies DOSS: Delirium Observation Screening Scale; CAM-ICU: Confusion Assessment Method Intensive Care Unit; EMR: electronic medical record, DSM: Diagnostic and Statistical Manual of Mental Disorders; SE: standard error; NOR: nonspecific outcomes reported; LT: liver transplant

Study	Population size	Delirium screening scale	Avg. SE	Indirectness	Other limitations	Quality of evidence
Densky et al., 2019 [14]	515	DSM-IV	0.3537	N/A	N/A	High
He and Fang, 2022 [15]	364	CAM-ICU	0.3961	N/A	N/A	High
Ma et al., 2021 [16]	325	CAM	0.4930	N/A	N/A	High
Şaşkın et al., 2016 [17]	935	CAM-ICU	0.4091	N/A	N/A	High
Bhattacharya et al., 2017 [18]	144	DOSS	0.0159	NOR	LT population	Moderate
Blinda et al., 2017 [19]	123	CAM-ICU	0.6028	N/A	LT population	Moderate
Chen et al., 2020 [20]	159	CAM-ICU	0.5158	N/A	LT population	Moderate
Chen et al., 2024 [21]	103	CAM	0.6738	NOR	N/A	Moderate
Crawford et al., 2021 [22]	1006	EMR mention of delirium	0.2754	N/A	N/A	Moderate
Guo et al., 2016 [23]	572	CAM	0.0198	N/A	Age >65 years	Moderate
Guo et al., 2017 [24]	385	CAM	0.7977	N/A	Burn Population	Moderate
Imai et al., 2024 [25]	192	CAM	0.9377	N/A	N/A	Moderate
Kuroki et al., 2019 [26]	112	DSM-IV	0.6460	N/A	N/A	Moderate
Lee et al., 2018 [27]	253	CAM-ICU	0.4583	N/A	LT population	Moderate
Li J. et al., 2021 [28]	1,426	CAM-ICU	0.2044	N/A	Age >65 years	Moderate
Li Y. et al., 2021 [29]	222	CAM	0.3644	NOR	Age >65 years	Moderate
Lin et al., 2023 [30]	692	CAM-ICU	0.3311	NOR	N/A	Moderate
Lu et al., 2022 [31]	402	CAM-ICU	0.4295	N/A	LT population	Moderate
Ma et al., 2023 [32]	321	CAM-ICU	0.3375	N/A	LT population	Moderate
Mou et al., 2023 [33]	863	DSM-V	0.2562	N/A	Age >65 years	Moderate
Oliver et al., 2017 [34]	181	EMR mention of delirium	0.4635	N/A	LT population	Moderate
Ooms et al., 2023 [35]	377	CAM-ICU	1.0894	N/A	N/A	Moderate
Park et al., 2017 [36]	196	DSM-V	0.5026	N/A	N/A	Moderate
Park et al., 2020 [37]	325	DSM-IV/ CAM	0.3322	N/A	LT population	Moderate
Patrono et al., 2020 [38]	309	CAM	0.3931	N/A	LT population	Moderate
Ri et a.l, 2020 [39]	260	DSM-IV	0.4781	NOR	LT population	Moderate
Shin et al., 2018 [40]	99	CAM-ICU	1.0100	N/A	N/A	Moderate
Broeke et al., 2018 [41]	329	DOSS	0.5567	N/A	N/A	Moderate
Wang et al., 2024 [42]	208	CAM-ICU	0.5000	N/A	Age >65 years	Moderate
Wu et al., 2021 [43]	228	CAM-ICU	0.4715	NOR	N/A	Moderate
Zhang et al., 2024 [44]	213	DSM-V	0.7449	N/A	N/A	Moderate
Cao et al., 2024 [45]	80	CAM	1.1034	NOR	N/A	Low
Chang et al., 2022 [46]	84	CAM-ICU	0.6030	N/A	LT population	Low
Spiropoulou et al., 2022 [47]	86	CAM-ICU	1.2502	N/A	Age >65 years	Low

Meta-Analysis

Multivariate ORs were isolated from the included studies. Inverse variance meta-analysis was performed within ReviewManager 5.4.1. Adjusted ORs were pooled for associations with POD under a random effects model based on a 95% confidence interval (CI). Two-sided p-values less than 0.05 were considered statistically significant. Pooled adjusted ORs for associations between indicators of preoperative hepatic impairment and POD were tabulated.

Publication Bias Assessment

Publication bias was evaluated visually using a funnel plot generated in Review Manager (RevMan) version 5.4. For the generic inverse variance meta-analysis, study-specific effect estimates and their standard errors were plotted on the horizontal and vertical axes, respectively. A pooled effect estimate was calculated using inverse-variance weighting, and a triangular 95% confidence region was overlaid. Symmetry of the plot was assessed qualitatively to identify potential small-study effects or missing studies. Egger’s regression test was performed by regressing the standard normal deviate of each study’s effect estimate against its precision (1/SE). A significant non-zero intercept was interpreted as evidence of small-study effects.

**Figure 2:**
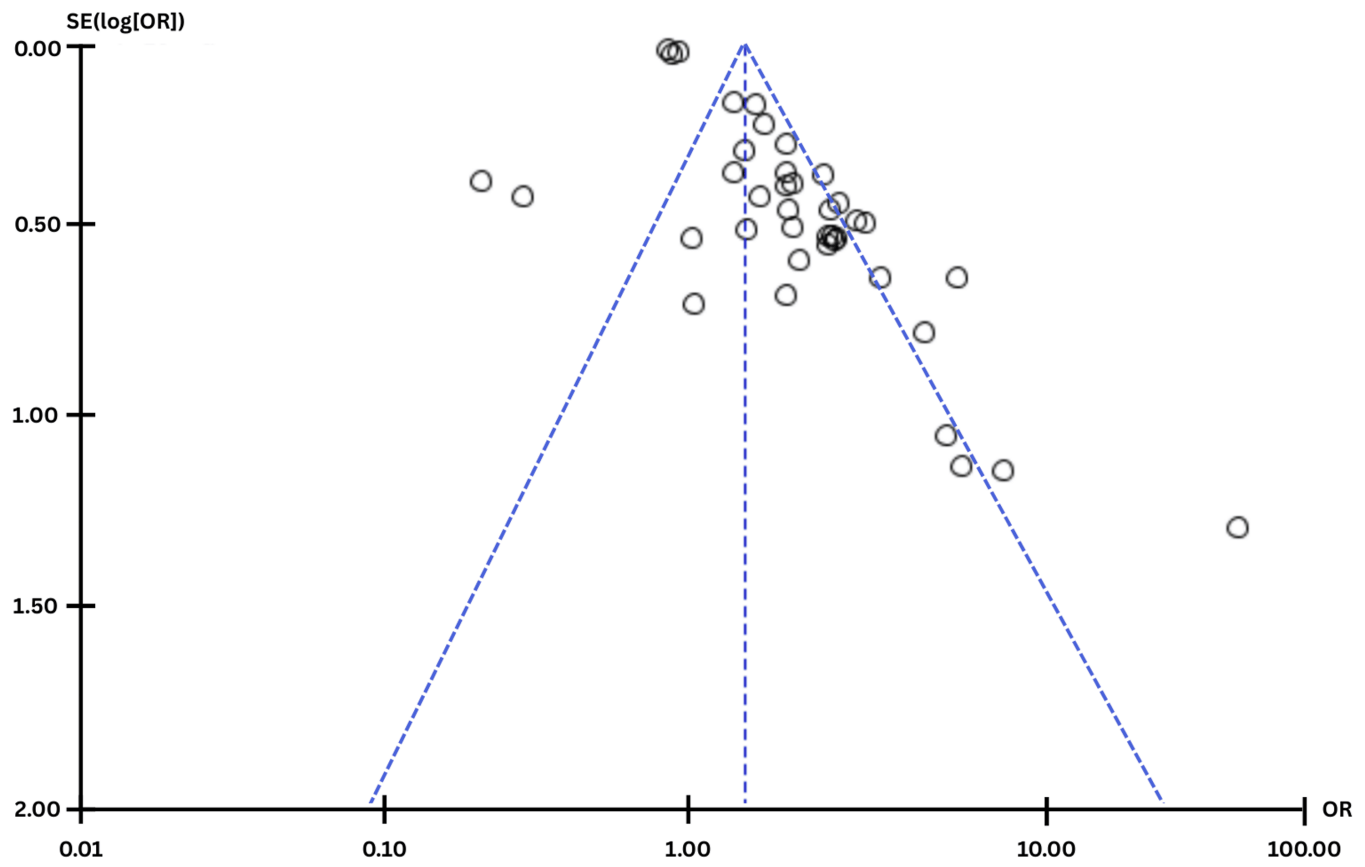
Funnel plot assessing for publication bias This figure depicts the funnel plot generated from the summary statistic used to assess for publication bias. Egger's test intercept for the summary statistic dataset was 1.92 with a p-value of 0.79, indicating a low risk for publication bias in the meta-analysis SE: standard error; OR: odds ratio

Results

Summary of Results

A total of 12,089 patients were included in this review, of which 2,663 developed POD, indicating an overall incidence of 22.03%. In total, nine different indicators of preoperative hepatic impairment were included in the review, namely: presence of anti-HCV antibodies, history of alcoholic liver disease, history of alcohol use, history of hepatic encephalopathy, elevated total bilirubin >3.3 mg/dL, elevated serum IL-6, elevated MELD score, elevated preoperative ammonia >46 umol/L, and alcohol abstinence within three months of undergoing general anesthesia (Table 2).

**Table 2:** Summary table GI: gastrointestinal; LT: liver transplantation; HENT: head, ears, nose, throat; CT: cardiothoracic; HCV: hepatitis C virus; MELD: Model for End Stage Liver Disease, OR: odds ratio; CI: confidence interval

Study	Study design	Surgical population	Population size	Incidence of POD (%)	Variable assessed	OR	95% CI	P-value
Park et al., 2017 [36]	Retrospective observational cohort study	GI	196	0.224	Anti-HCV antibody-positive	1.181	0.441 - 3.164	0.7406
Park et al., 2020 [37]	Retrospective observational cohort study	LT	325	0.212	Alcoholic liver disease	1.63	0.850 - 3.127	0.141
Chen et al., 2024 [21]	Prospective randomized and double-blinded controlled trial	GI	103	0.184	History of alcohol use	1.195	0.319 - 4.476	0.791
Crawford et al., 2021 [22]	Retrospective observational cohort study	HENT	1006	0.075	History of alcohol use	1.75	1.02 - 23.0	0.0422
Guo et al., 2017 [24]	Retrospective observational cohort study	Burn	385	0.145	History of alcohol use	19.34	4.05 - 92.25	0.0002
He and Fang, 2022 [15]	Retrospective observational cohort study	CT	364	0.255	History of alcohol use	0.326	0.15 - 0.71	0.706
Imai et al., 2024 [25]	Retrospective observational cohort study	HENT	192	0.224	History of alcohol use	2.554	1.260 - 5.268	0.0098
Kuroki et al., 2019 [26]	Retrospective observational cohort study	GI	112	0.277	History of alcohol use	2.405	0.678 - 8.534	0.175
Li J. et al., 2021 [28]	Retrospective observational cohort study	CT	1,426	0.393	History of alcohol use	2.06	1.38 - 3.10	<0.001
Li Y. et al., 2021 [29]	Prospective observational cohort study	GI	222	0.41	History of alcohol use	2.398	1.174 - 4.900	0.016
Ma et al., 2021 [16]	Retrospective observational cohort study	Orthopedic	325	0.178	History of alcohol use	3.414	1.299 - 8.975	0.013
Mou et al., 2023 [33]	Retrospective observational cohort study	Neurosurgery	863	0.1	History of alcohol use	2.427	1.469 - 3.384	0.011
Ooms et al., 2023 [35]	Retrospective observational cohort study	HENT	377	0.106	History of alcohol use	9.22	1.09 - 77.97	0.041
Saskin et al., 2016 [17]	Retrospective observational cohort study	CT	935	0.225	History of alcohol use	3.59	1.61 - 7.98	0.002
Shin et al., 2018 [40]	Retrospective observational cohort study	Orthopedic	99	0.404	History of alcohol use	8.18	1.13 - 16.60	0.0374
Spiroopoulou et al., 2022 [47]	Retrospective observational cohort study	CT	86	0.256	History of alcohol use	74.3	6.41 - 861	0.0006
Broeke et al., 2018 [41]	Prospective observational cohort study	CT	329	0.128	History of alcohol use	2.65	0.89 - 7.90	0.08
Wang et al., 2024 [42]	Retrospective observational cohort study	Neurosurgery	208	0.524	History of alcohol use	3.549	1.332 - 9.459	0.0113
Zhang et al., 2024 [44]	Retrospective observational cohort study	Neurosurgery	213	0.296	History of alcohol use	6.89	1.60 - 29.68	0.01
Lin et al., 2023 [30]	Retrospective observational cohort study	CT	692	0.318	History of alcohol use	2.407	1.258 - 3.608	0.008
					Total bilirubin elevated	1.907	1.402 - 2.513	0.0001
					Serum IL-6	1.616	1.210 - 2.022	0.0011
Blinda et al., 2017 [19]	Retrospective observational cohort study	LT	123	0.122	History of hepatic encephalopathy	8.8	2.7 - 28.59	0.0003
Chen et al., 2020 [20]	Retrospective observational cohort study	LT	159	0.264	History of hepatic encephalopathy	3.298	1.200 - 9.065	0.021
					MELD ≥15	3.334	1.265 - 8.786	0.015
					Preoperative ammonia >46 umol/L	3.513	1.306 - 9.455	0.013
Oliver et al., 2017 [34]	Retrospective observational cohort study	LT	181	0.21	History of hepatic encephalopathy	4.39	1.77 - 10.9	0.0014
Patrono et al., 2020 [38]	Retrospective observational cohort study	LT	309	0.133	History of hepatic encephalopathy	1.988	0.920 - 4.296	0.8
Cao et al., 2024 [45]	Retrospective observational cohort study	Neurosurgery	80	0.263	IL-6	12.659	1.456 - 110.082	0.021
Bhattacharya et al., 2017 [18]	Retrospective observational cohort study	LT	144	0.25	MELD	0.98	0.95 - 0.99	0.2028
Lee et al., 2018 [27]	Retrospective observational cohort study	LT	253	0.17	MELD ≥15	4.1	1.67 - 10.09	0.002
Ma et al., 2023 [32]	Retrospective observational cohort study	LT	321	0.193	MELD >22	3.4	1.468 - 7.876	0.004
					History of hepatic encephalopathy	3.224	1.664 - 6.244	0.001
Ri et al., 2020 [39]	Retrospective observational cohort study	LT	260	0.142	MELD	1.02	0.967 - 1.076	0.458
					History of alcohol use	1.792	0.702 - 4.573	0.222
Chang et al., 2022 [46]	Retrospective observational cohort study	LT	84	0.548	Preop alcohol abstinence <3 months	4.953	1.519 - 16.152	0.008
Densky et al., 2019 [14]	Retrospective observational cohort study	HENT	515	0.109	Preop alcohol abstinence <3 months	0.24	0.12 - 0.51	0.0001
Guo et al., 2016 [23]	Prospective observational cohort study	Orthopedic	572	0.21	Total bilirubin >14 umol/L	1.077	1.036 - 1.121	0.002
Lu et al., 2022 [31]	Retrospective observational cohort study	LT	402	0.194	Total bilirubin >3.3 mg/dL	2.46	1.06 - 5.69	0.035
Wu et al., 2021 [43]	Prospective observational cohort study	Non-CT	228	0.25	Total or direct bilirubin elevated	2.535	1.006 - 6.388	0.0485

POD in Different Surgical Populations

**Figure 3:**
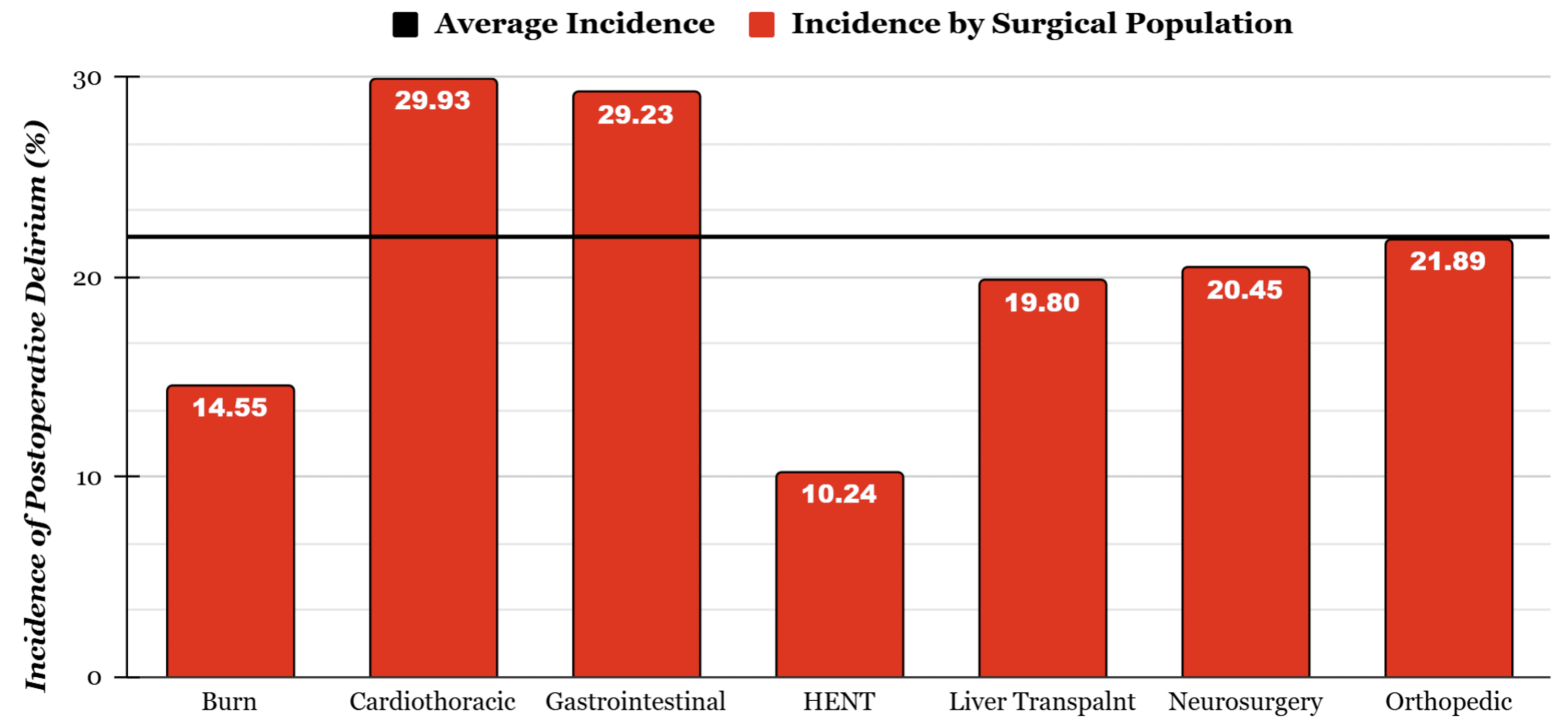
Incidence of POD in different surgical populations This figure includes the incidence of POD by the surgical population weighted by population size. Burn - [24]; cardiothoracic - [15,17,28,30,41,47]; gastrointestinal - [21,26,29,36]; HENT- [14,22,25,35]; liver transplant - [18,19,20,27,31,32,34,37,38,39,46]; neurosurgery - [33,42,44,45]; orthopedic - [16,23,40] POD: postoperative delirium; HENT: head, ears, nose, throat

Across the 34 included studies, seven different surgical populations were included: liver transplantation, gastrointestinal (other than liver transplantation), cardiothoracic, head-ears-nose-throat, orthopedic, neurosurgery, and burn populations. The incidence of delirium was the highest in the cardiothoracic population at 29.93% and the lowest in the head, ears, nose, throat (HENT) population at 10.24% (Figure 3).

Meta-Analysis Results

**Figure 4:**
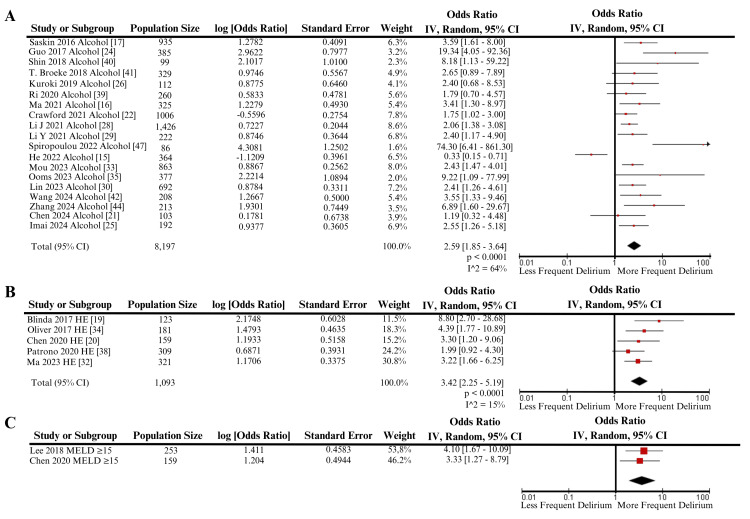
Forest plots of meta-analysis examining the association between alcohol use (A), hepatic encephalopathy (B), and MELD ≥15 with postoperative delirium risk This figure depicts the forest plots for significant meta-analyses. Panel A: forest plot examining the association between alcohol use and postoperative delirium. Panel B: forest plot examining the association between HE (hepatic encephalopathy) and postoperative delirium. Panel C: forest plot examining the association between MELD ≥15 and postoperative delirium IV: inverse variance; MELD: Model for End-Stage Liver Disease; CI: confidence interval

Figure 4 is a forest plot that displays the significant outcomes resulting from inverse variance meta-analysis. Three hepatic variables met statistical significance: history of alcohol use (OR: 2.59, 1.85 - 3.64, p<0.0001), history of hepatic encephalopathy (OR: 3.42, 2.25 - 5.19, p<0.0001) (Table 3), and MELD ≥15 (OR: 3.73, 1.93 - 7.20, p<0.0001). Two other variables were analyzed by meta-analysis but did not find significance: elevated serum IL-6 (OR: 3.38, 0.49 - 23.43, p=0.22), and elevated total bilirubin (OR: 1.57, 0.94 - 2.63, p=0.09) (Table 3).

**Table 3:** Meta-analysis results This table includes the results of inverse variance random-effects meta-analysis OR: odds ratio; CI: confidence interval; IL-6: interleukin-6; MELD: Model for End-Stage Liver Disease

Outcome assessed	Pooled adjusted OR	95% CI	P-value	I^2^
History of alcohol use	2.59	1.85 - 3.64	<0.0001	64%
History of hepatic encephalopathy	3.42	2.25 - 5.19	<0.0001	15%
Elevated serum IL-6	3.38	0.49 - 23.43	0.22	71%
MELD score ≥15	3.73	1.93 - 7.20	<0.0001	0%
Elevated total bilirubin	1.57	0.94 - 2.63	0.09	88%

Discussion

The goal of this systematic review and meta-analysis was to identify the role that specific indicators of preoperative hepatic impairment contribute to increased risk for POD. We aimed to identify the incidence rate of POD in different surgical populations. To the knowledge of the authors of this review, there are no similar reviews in the literature that address this research question. The final results of our study reveal that known indicators of hepatic impairment in a patient’s past medical history, namely alcohol use and previous diagnosis of hepatic encephalopathy, are significant factors for POD (Table 3). Also, a MELD score ≥15 was found to be a risk factor. Specifically, patients with a history of alcohol use were 2.59 times more likely to develop POD, whereas a history of hepatic encephalopathy carried a 3.42 times increased risk (Table 3), and a MELD score ≥15 carried a 3.73 times increased risk.

A history of hepatic encephalopathy is a logical risk factor for POD, as the most significant risk factor for POD in general is preoperative cognitive dysfunction [48]. While hepatic encephalopathy can present with varying symptoms, the most common are confusion, forgetfulness, and slurred speech, all of which coincide with delirium [49]. The symptoms of hepatic encephalopathy are largely attributed to elevated ammonia levels [50, 51]. Only one study reported a multivariate odds ratio for preoperative ammonia level > 46 umol/L, which found significance and reported an odds ratio of 3.513 (CI 1.306 - 9.455) for association with POD [20]. Overall, alcohol and hepatic encephalopathy are significant risk factors for POD, and care should be taken to mitigate other factors that may put these predisposed patients at further risk.

There are a few limitations to this study that should be addressed. First, our systematic review was specific in its inclusion criteria. Specifically, we required multivariate odds ratios with corresponding 95% confidence intervals and p-values for inclusion. Our goal was to synthesize a focused review, which may have led to some tangentially beneficial studies being excluded. The quality assessment cut-off values for the average standard error and population size, although consistent, are rigidly selected. They did not differentiate studies that were very close to satisfying the requirement from those that were not. One of the more common etiologies of POD is active infection, which was not controlled for in this review.

Furthermore, some studies were nonspecific in the reporting of their outcomes, as outlined in Table 1. This is especially true for the studies that reported a history of alcohol use, as this is an extremely difficult metric to standardize. This likely contributed to substantial heterogeneity between meta-analyzed studies when pooling nonspecific outcomes. Lastly, an elevated MELD score ≥15 was found to be a significant risk factor; however, only two studies were included that utilized this stringent cut-off. Further research should be conducted to analyze the role MELD scoring may play in POD prediction. Confounding effects were minimized by requiring multivariate odds ratios and utilizing an inverse variance meta-analysis.

## Conclusions

Based on the results of this study, it is evident that individuals with a known medical history of hepatic pathology are at increased risk of developing POD. Namely, alcohol consumption and history of hepatic encephalopathy were both found to be statistically significant contributors to POD. However, specific biomarkers (elevated bilirubin and serum IL-6) linked to hepatic function did not show significance in our study. Likewise, an elevated MELD score did not meet statistical significance in contributing to POD. While specific hepatic biomarkers were not necessarily implicated in POD, further research in quantifying the effects of lifestyle factors (consumption of alcohol) and history of gross liver impairment (hepatic encephalopathy) is warranted to better understand how overall liver function affects postoperative outcomes when general anesthesia is required.

## Appendices

**Table 4:** Search string used across all databases This table depicts the entire search string utilized to extract candidate studies from Embase, Web of Science, and PubMed

Embase - 791 results	
1.	'delirium*':ti,ab,kw OR 'confusion*':ti,ab,kw OR 'altered mental status*':ti,ab,kw
2.	'postoperative*':ti,ab,kw OR 'postoperative period*':ti,ab,kw OR 'postoperative complication*':ti,ab,kw
3.	'hepatic*':ti,ab,kw OR 'liver*':ti,ab,kw OR 'aspartate aminotransferase*':ti,ab,kw OR 'ast*':ti,ab,kw OR 'alanine aminotransferase*':ti,ab,kw OR 'alt*':ti,ab,kw OR 'alkaline phosphatase*':ti,ab,kw OR 'alp*':ti,ab,kw OR 'gamma-glutamyl transferase*':ti,ab,kw OR 'ggt*':ti,ab,kw OR 'alpha-fetoprotein*':ti,ab,kw OR 'afp*':ti,ab,kw OR 'ceruloplasmin*':ti,ab,kw OR 'nucleotidase*':ti,ab,kw OR 'ntp*':ti,ab,kw OR 'bilirubin*':ti,ab,kw OR 'cirrhosis*':ti,ab,kw OR 'alcohol*':ti,ab,kw OR 'alcoholic*':ti,ab,kw OR 'hepatitis*':ti,ab,kw OR 'hepatocellular carcinoma*':ti,ab,kw OR 'hcc*':ti,ab,kw OR 'hemochromatosis*':ti,ab,kw
4.	#1 AND #2 AND #3
5.	#4 AND (2015:py OR 2016:py OR 2017:py OR 2018:py OR 2019:py OR 2020:py OR 2021:py OR 2022:py OR 2023:py OR 2024:py OR 2025:py) AND 'article'/it

MEDLINE-PubMed - 193 results	
1.	"delirium"[Title/Abstract] OR "confusion"[Title/Abstract] OR "altered mental status"[Title/Abstract]
2.	“postoperative"[Title/Abstract] OR "postoperative period"[Title/Abstract] OR "postoperative complication"[Title/Abstract]
3.	“hepatic"[Title/Abstract] OR “liver"[Title/Abstract] OR “aspartate aminotransferase"[Title/Abstract] OR “ast"[Title/Abstract] OR “alanine aminotransferase"[Title/Abstract] OR “alt"[Title/Abstract] OR “alkaline phosphatase"[Title/Abstract] OR “alp"[Title/Abstract] OR “gamma-glutamyl transferase"[Title/Abstract] OR “ggt"[Title/Abstract] OR “alpha-fetoprotein"[Title/Abstract] OR “afp"[Title/Abstract] OR “ceruloplasmin"[Title/Abstract] OR “nucleotidase"[Title/Abstract] OR “ntp"[Title/Abstract] OR “bilirubin"[Title/Abstract] OR “cirrhosis"[Title/Abstract] OR “alcohol"[Title/Abstract] OR “alcoholic"[Title/Abstract] OR “hepatitis"[Title/Abstract] OR “hepatocellular carcinoma"[Title/Abstract] OR “hcc"[Title/Abstract] OR “hemochromatosis"[Title/Abstract]
4.	#1 AND #2 AND #3
5.	(#4) 10 years

Web of Science - 243 results	
1.	TS=(delirium OR confusion OR altered mental status)
2.	TS=(postoperative OR postoperative period OR postoperative complication)
3.	TS=(hepatic OR liver OR aspartate aminotransferase OR ast OR alanine aminotransferase OR alt OR alkaline phosphatase OR alp OR gamma-glutamyl transferase OR ggt OR alpha-fetoprotein OR afp OR ceruloplasmin OR nucleotidase OR ntp OR bilirubin OR cirrhosis OR alcohol OR alcoholic OR hepatitis OR hepatocellular carcinoma OR hcc OR hemochromatosis)
4.	#1 AND #2 AND #3
5.	(#4) 2015-2025
